# Hierarchical porous carbons with layer-by-layer motif architectures from confined soft-template self-assembly in layered materials

**DOI:** 10.1038/ncomms15717

**Published:** 2017-06-12

**Authors:** Jie Wang, Jing Tang, Bing Ding, Victor Malgras, Zhi Chang, Xiaodong Hao, Ya Wang, Hui Dou, Xiaogang Zhang, Yusuke Yamauchi

**Affiliations:** 1 Key Laboratory of Materials and Technologies for Energy Conversion, College of Materials Science & Engineering, Nanjing University of Aeronautics and Astronautics, Nanjing 210016, China; 2 International Center for Materials Nanoarchitectonics (MANA), National Institute for Materials Science (NIMS), 1-1 Namiki, Tsukuba, Ibaraki 305-0044, Japan; 3 Faculty of Science and Engineering, Waseda University, 3-4-1 Okubo, Shinjuku, Tokyo 169-8555, Japan; 4 Australian Institute for Innovative Materials (AIIM), University of Wollongong, North Wollongong, New South Wales 2500, Australia

## Abstract

Although various two-dimensional (2D) nanomaterials have been explored as promising capacitive materials due to their unique layered structure, their natural restacking tendency impedes electrolyte transport and significantly restricts their practical applications. Herein, we synthesize all-carbon layer-by-layer motif architectures by introducing 2D ordered mesoporous carbons (OMC) within the interlayer space of 2D nanomaterials. As a proof of concept, MXenes are selected as 2D hosts to design 2D–2D heterostructures. Further removing the metal elements from MXenes leads to the formation of all-carbon 2D–2D heterostructures consisting of alternating layers of MXene-derived carbon (MDC) and OMC. The OMC layers intercalated with the MDC layers not only prevent restacking but also facilitate ion diffusion and electron transfer. The performance of the obtained hybrid carbons as supercapacitor electrodes demonstrates their potential for upcoming electronic devices. This method allows to overcome the restacking and blocking of 2D nanomaterials by constructing ion-accessible OMC within the 2D host material.

## Abstract (web summary)

2D nanomaterials are promising capacitive energy storage materials, but their tendency to restack hinders electrolyte transport. Here, Yamauchi and colleagues introduce 2D ordered mesoporous carbons in between MXene layers, and metal removal affords all-carbon porous 2D–2D heterostructures in which restacking is prevented.

Two-dimensional (2D) nanomaterials, which are composed of single- or few-atom thick nanosheets, are a current research hotspot for diverse applications due to their recognized intrinsic properties (for example, high specific surface area (SSA), flexible lamellar channel and tunable electronic structure)^1^,^2^,^3^,^4^,^5^. A newer family of 2D nanosheets, MXenes, were first obtained by Gogotsi in 2011 from the chemical etching and delamination of their MAX layered counterparts^6^. The MAX phase is a group of layered ternary carbides and nitrides with formula M_*n*+1_AX_*n*_ (*n*=1, 2, 3), where M represents an early transition metal, A is an A-group element (mostly 13 or 14A-group), and X represents carbon or nitrogen^7^,^8^,^9^. After selective extraction of the A element from MAX with hydrofluoric acid (HF), 2D layered nanomaterials M_*n*+1_X_*n*_T_*x*_ (*n*=1, 2, 3) can be obtained, named as MXenes to highlight their structural similarity with graphene. In the chemical formula, T represents the hydroxyl (−OH) and/or fluorine (-F) surface terminal groups, and *x* indicates the number of the surface groups introduced during HF etching^6^,^7^,^10^,^11^.

MXenes possess a unique metallic electrical conductivity (for example, 2.4 × 10^5^ S m^−1^ for Ti_3_C_2_T_*x*_ film)^12^, which is far better than that of other well-known 2D nanomaterials such as transition metal oxide/dichalcogenide, layered double-hydroxide, or even graphene. Thus, the hydrophilic MXenes have been manufactured as electrode materials for capacitive energy storage and showed extraordinary volumetric capacitance^3^,^13^,^14^,^15^, which is even higher than for the best carbon materials^16^,^17^. However, the common shortcomings in 2D nanomaterials, especially the natural restacking tendency, have not been fully addressed as yet, thus limiting the implementation of 2D nanomaterials such as MXenes in practical applications^18^.

One possible method to prevent the restacking of nanosheets is to prepare crumpled or curved nanosheets, which creates spacings when packed together^19^; however, the accessibility of the parallel channels is impaired. Another strategy is to grow 2D nanosheets vertically aligned to the substrate by chemical vapour deposition methods^20^,^21^; although the parallel planar channels are well-maintained, the deposited amount of 2D nanosheets per unit area is restricted, thus limiting the performance. The third approach consists in incorporating small dimensional nanoparticles or molecules (for example, 0D carbon onions^22^ or quantum dots^23^, 1D carbon nanotubes^18^,^24^, 2D carbon nanosheets^25^,^26^, and polymers^12^,^27^) within the interlayers, acting as spacers between the 2D nanosheets. Most of the heterostructures are built on van der Waals forces which allow the free integration of 2D nanosheets with disparate nanomaterials. However, this strategy results in the nondirective and non-uniformly distributed interlayer spacers in host materials, which leads to a complex interlayer pathway for ion diffusion and/or electron transfer. Thus, it is still a great challenge to build regular, rigid, porous and electron conductive bridges between the nanosheets, while preventing their restacking and promoting mass transportation.

Herein, as a proof of concept, we fabricate 2D–2D heterostructures by designing the intercalation of 2D ordered mesoporous carbon (OMC) thin layers within the MXenes interlayer spaces. The 2D OMC intercalated between the MXenes layers not only prevents the restacking of the nanosheets but the highly interconnected nanoporous network also provides accessible pathways for ion diffusion while maintaining fast electron transfer. The concept proposed here will open a new paradigm for the synthesis of 2D hybrid nanomaterials and broaden their range of applications.

## Results

### Formation process and materials characterization

**Figure 1:**
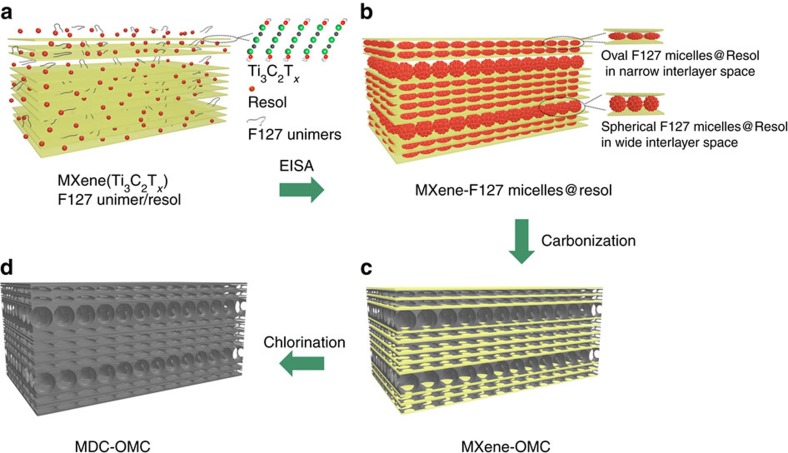
Schematic illustration of the synthetic route. Preparation of the (**a**) MXene/F127 unimer/resol mixture, (**b**) MXene-F127 micelles@resol composite, (**c**) MXene-OMC composite and (**d**) MDC-OMC composite.

The synthetic route is illustrated in Fig. 1. Ti_3_C_2_T_*x*_ is selected as a representative example of 2D layered MXenes, considering its well-explored exfoliation process from the parent Ti_3_AlC_2_. Amphiphilic triblock copolymer F127 (PEO-PPO-PEO) and pre-synthesized low-molecular-weight phenolic resols are added to a dispersion of Ti_3_C_2_T_*x*_ in ethanol. The well-dissolved F127 unimers and the small phenolic resol molecules can easily penetrate the interlayers of Ti_3_C_2_T_*x*_ (Fig. 1a). Afterwards, the well-mixed solution is placed in a vacuum drying oven to evaporate the ethanol and compel the intercalation of F127 unimers and resol molecules inside the interlayers of Ti_3_C_2_T_*x*_. Owing to the gradually evaporating ethanol, the F127 unimers are induced to assemble into micelles, composed of hydrophobic PPO block as the cores and hydrophilic PEO block (with -OH terminal groups) as the shells, which interact with the resol molecules through hydrogen bonding. As shown in Fig. 1b, the continuously evaporating ethanol leads to the close-packing assembly of micelle@resol and the formation of 2D ordered micelle@resol layers between the Ti_3_C_2_T_*x*_ nanosheets. Thus, Ti_3_C_2_T_*x*_-micelle@resol composites are obtained (Fig. 1b). After thermal treatment of Ti_3_C_2_T_*x*_-micelle@resol composites under inert atmosphere (Fig. 1c), the introduced micelle@resol is converted into OMC layers, forming a 2D–2D heterostructure (abbreviated as Ti_3_C_2_T_*x*_-OMC). After etching the metal from the host MXene material, the MXene-derived carbons (MDC) consisting of microporous carbon nanosheets can be obtained. Thus, all-carbon material, MDC-OMC, can be prepared by completely removing Ti from Ti_3_C_2_T_*x*_-OMC through chlorination (Fig. 1d).

**Figure 2:**
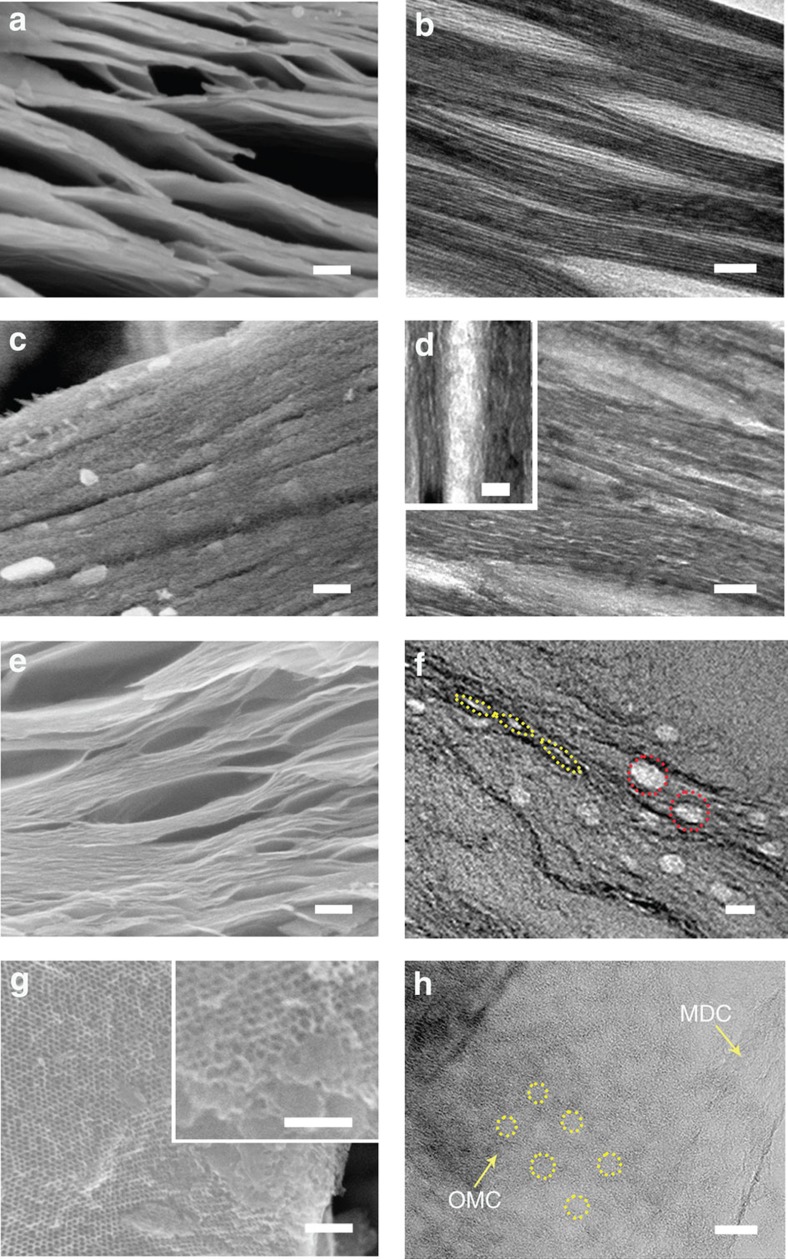
Morphology characterization. (**a**) Cross-section SEM and (**b**) TEM images of the Ti_3_C_2_T_*x*_. (**c**) Cross-section SEM and (**d**) TEM images of the Ti_3_C_2_T_*x*_-OMC. (**e**) Cross-section SEM and (**f**) TEM images of the MDC-OMC. (**g**) Top-view SEM and (**h**) TEM images of the MDC-OMC. Scale bars are 100 nm for **a**,**c**,**e**,**g**, inset in **g**, and 20 nm for **b**,**d**, inset in **d**,**f**, **h**.

The above formation process based on the intercalation of micelle@resol can be examined by scanning electron microscope (SEM) and transmission electron microscope (TEM). Figure 2 shows SEM and TEM images of the Ti_3_C_2_T_*x*_ host material, Ti_3_C_2_T_*x*_-OMC and MDC-OMC. The SEM and TEM images in Fig. 2a,b show the typical morphology of 5–10 layer thick Ti_3_C_2_T_*x*_ bundles. Two types of spacing can be observed: the interlayer space (∼2 nm), and the much larger space between the bundles (∼10–20 nm). After intercalating the close-packing assembly of micelle@resol within the Ti_3_C_2_T_*x*_ and further carbonization, the obtained Ti_3_C_2_T_*x*_-OMC exhibits thicker and rougher layers (Fig. 2c) compared to the original Ti_3_C_2_T_*x*_ host (Fig. 2a), which is due to the inserted mesoporous carbon layers. The inset TEM image in Fig. 2d shows the aligned mesopores within the large space between the bundles. We can also anticipate the presence of several pores in the interlayer space.

After completely etching the metal (Ti) from the host Ti_3_C_2_T_*x*_ material through chlorination, the MDC-OMC (Fig. 2e,f and Supplementary Fig. 1) clearly shows the layered structure and two types of mesopores (as indicated by the circles): one is spherical and located within the large space between the bundles (red circle), and the other is oval and located within the interlayer spaces (yellow circle). Based on this result, we can assume that two possible mechanisms occur during the solvent evaporation process under vacuum conditions (Fig. 1b). In the large space between the bundles, the F127 unimers easily assemble into spherical micelles and interact with the resol molecules through hydrogen bonding to finally assemble into single-layered or multi-layered micelle@resol mesostructures. In contrast, in the narrow interlayer space, the micelle@resol molecules are confined and tightly aggregated, leading to the formation of ellipsoidal mesostructures. The forces driving the intercalation inside the interlayer space probably originate from the strong interaction between micelle@resol subunits (rich in phenolic hydroxyl groups) and the surface of the Ti_3_C_2_T_*x*_ nanosheets (rich in –OH and –F terminal groups). After the aggregation and crosslinking of the resol molecules, the micelle@resol are successively transformed into mesostructured polymers. Then, high-temperature calcination decomposes the micelles and finally generates two types of pores (spherical and oval). We consider that the micelle@resol assemblies tend to be single-layer (at most a few layers), due to the confined interlayer space of the Ti_3_C_2_T_*x*_ host material, as shown in Fig. 2d. As observed on the cross-section TEM images (Supplementary Fig. 1), the longitudinal diameters of mesopores are almost 15 nm while the shorter diameters are determined by the interlayer spacing of Ti_3_C_2_T_*x*_. Top-view SEM and TEM images of the MDC-OMC further confirms that the mesopores with diameter of ∼10–15 nm are arranged in a 2D hexagonal ordering over large domains of several micrometers on the same layer (Fig. 2g,h and Supplementary Fig. 2). The average pore wall thickness in the OMC is estimated to be 2 nm, which is consistent with previously published results^28^,^29^,^30^. For comparison, the MDC without OMC was prepared and the SEM and TEM images (Supplementary Fig. 3) show a totally different porous structure, mainly consisting of slit-like pores between the layers. However, due to the ultrathin and alternating MDC and OMC nanosheets, the TEM image can hardly resolve the inner mesopore arrays.

**Figure 3:**
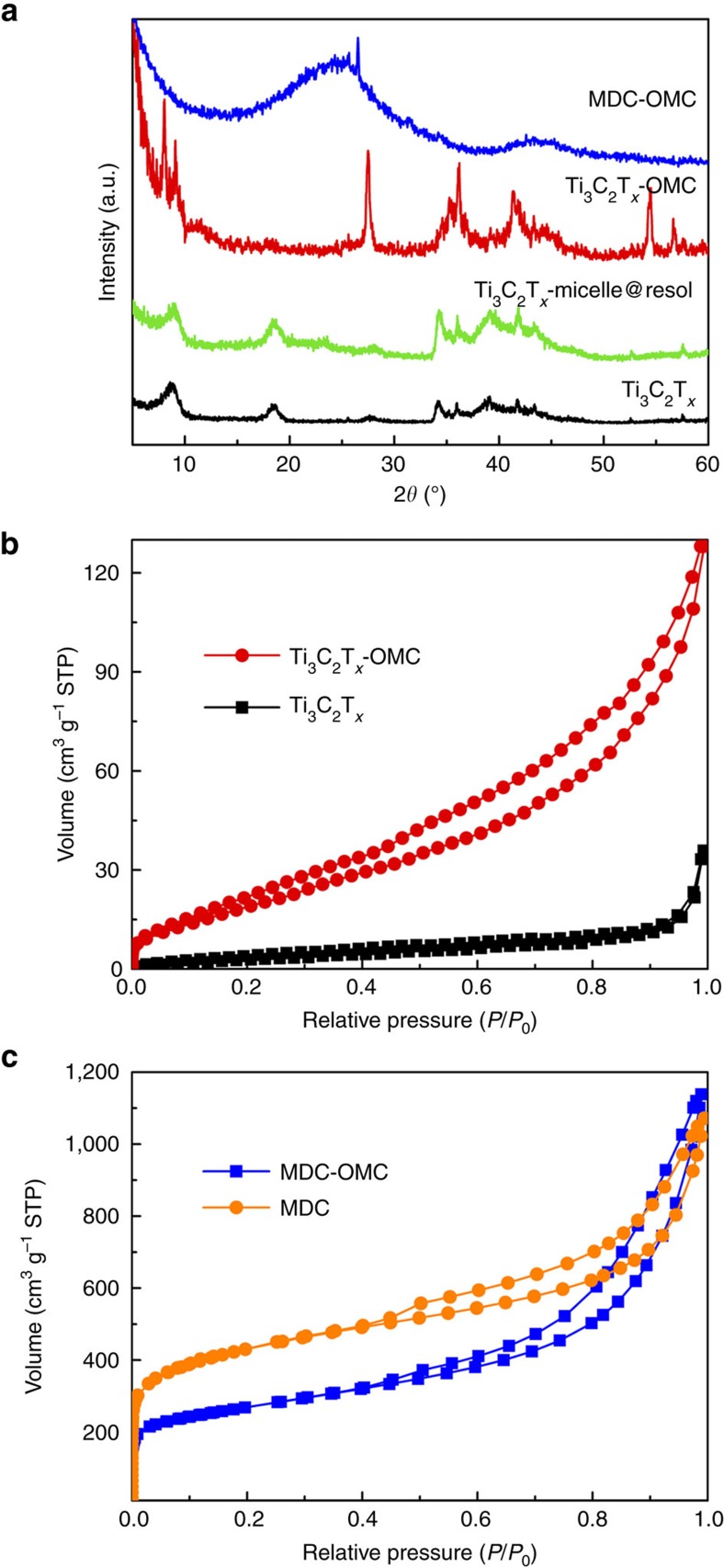
Evidence of OMC intercalation. (**a**) XRD patterns of Ti_3_C_2_T_*x*_, Ti_3_C_2_T_*x*_-micelle@resol, Ti_3_C_2_T_*x*_-OMC and MDC-OMC. Nitrogen adsorption–desorption isotherms of (**b**) Ti_3_C_2_T_*x*_ and Ti_3_C_2_T_*x*_-OMC and (**c**) MDC and MDC-OMC.

X-ray diffraction (XRD) provides further information for the above intercalation process (Fig. 3a). After introducing the micelle@resol, the diffraction peaks of the Ti_3_C_2_T_*x*_-micelle@resol at ∼18° and ∼33–45°, as well as the (0002) interlayer spacing distance at ∼9°, remain unchanged, implying the steady structures of Ti_3_C_2_T_*x*_ host. Upon carbonization, the (0002) peak is clearly downshifted to lower angle and distributed into several peaks, suggesting the different interlayer spaces by locations (Fig. 2d). After high temperature chlorination, distortion of the regular interlayer spaces occurs, resulting in small ordered mesostructural domains. Therefore, we cannot observe obvious diffraction peaks in the low-angle XRD pattern of MDC-OMC^29^,^30^. No residual carbide can be identified from the XRD pattern of MDC-OMC (Fig. 3a), indicating that the Ti_3_C_2_T_*x*_ in Ti_3_C_2_T_*x*_-OMC is fully converted into pure carbon materials. The two XRD peaks of MDC-OMC centred at 24° and 44° can be attributed to the (002) and (101) crystallographic planes of graphite, respectively. The broad reflections peaks are characteristic for disordered carbon. Raman spectroscopy (Supplementary Fig. 4 and Supplementary Note 1) also confirms the coexistence of disordered carbon and ordered graphitic carbon in the MDC-OMC. After being determined by the inductively coupled plasma and thermogravimetric (TG) analysis (explained in detail in Supplementary Fig. 5 and Supplementary Note 2), the weight percentage of Ti_3_C_2_T_*x*_ and OMC is reckoned to be ∼85% and ∼15% in the Ti_3_C_2_T_*x*_-OMC composite, respectively. After chlorination, the percentage of MDC and OMC is about 38.4% and 61.6%, respectively, in MDC-OMC.

The porosity changes were also characterized by nitrogen adsorption–desorption experiments (Fig. 3b,c and Supplementary Fig. 6). In comparison to the Ti_3_C_2_T_*x*_ host material, the SSA and pore volume of the Ti_3_C_2_T_*x*_-OMC are significantly increased from 13 to 84 m^2^ g^−1^ and from 0.05 to 0.19 cm^3^ g^−1^, respectively. After chlorination, the SSA of the MDC-OMC is further increased up to 1,021 m^2^ g^−1^ while the SSA of MDC is calculated to be 1,536 m^2^ g^−1^. More precisely, the proportion of microporous surface area decrease from 58% in MDC to 36% in MDC-OMC, and the ratio of micro-pore volume are decreased from 32% in MDC to 10% in MDC-OMC (all data are summarized in Supplementary Table 1). Compared to MDC that shows a considerable amount of micropores, the MDC-OMC exhibits lower SSA due to the introduction of mesopores (that is, generally the SSA tends to decrease with increasing the pore size^31^). In addition, an important proportion of the in-plane micropores are expected to be sacrificed (where MDC and OMC are connected tightly) in MDC-OMC, leading to a decreased SSA. It is noteworthy that the SSA of the MDC-OMC is similar to the mixed sample of MDC/OMC (1,080 m^2^ g^−1^), which is prepared by simply mixing the same proportion of MDC and OMC as in the MDC-OMC. This result implies that the intercalated OMC layers do not block the interlayer channels in the MDC and provide extra mesopores in MDC-OMC. The micro- and meso-pores distributed in the MDC and OMC layers are interconnected, otherwise, nitrogen molecules would be obstructed and could not be adsorbed in the in-plane and interlayer pores. Supplementary Fig. 6 displays the analysis of the pore size distribution by applying the nonlocal density functional theory (NLDFT) method using one-dimensional slit pore model. The pore size distribution curves indicate that the MDC-OMC and MDC samples have similar pore sizes centred at 0.5, 1.2, 3.1 and 7.0–12.0 nm. This result suggests that the pore diameter of the monolayered mesoporous structure in the interlayer space cannot be assessed by the present NLDFT analysis, because of confined lamellar structure of the host MDC. In addition, the mesopore size distribution of MDC-OMC is similar to the OMC (Supplementary Fig. 7), which are centred at 2–5 nm.

### Electrochemical performance

**Figure 4:**
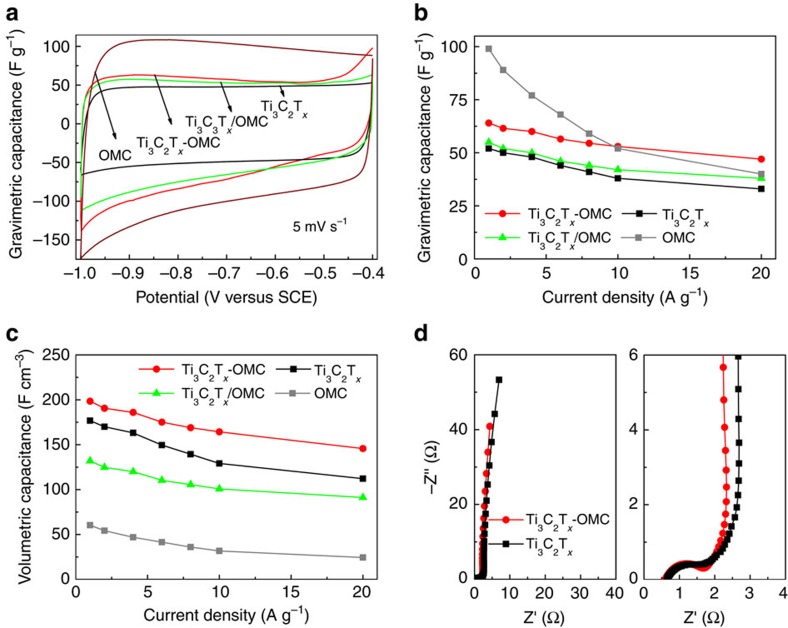
Electrochemical performance of the MXene-OMC. (**a**) CV curves of the Ti_3_C_2_T_*x*_-OMC, Ti_3_C_2_T_*x*_/OMC, Ti_3_C_2_T_*x*_ and OMC at a scan rate of 5 mV s^−1^. (**b**) Gravimetric capacitances versus different current densities and (**c**) volumetric capacitances versus different current densities for the Ti_3_C_2_T_*x*_-OMC, Ti_3_C_2_T_*x*_/OMC, Ti_3_C_2_T_*x*_ and OMC. (**d**) Nyquist plots of the Ti_3_C_2_T_*x*_-OMC and Ti_3_C_2_T_*x*_ with the close-up view on the high-frequency regime. The reference electrode is a saturated calomel electrode (SCE).

Owing to its unique structure as well as the inserted mesoporous carbon layers, the Ti_3_C_2_T_*x*_-OMC is expected to be a promising candidate for electrode materials for supercapacitor applications. Using a three-electrode electrochemical cell, the electrochemical properties of the Ti_3_C_2_T_*x*_-OMC electrode were investigated in a 6 M KOH electrolyte and summarized in Fig. 4. For comparison, the supercapacitive performance of Ti_3_C_2_T_*x*_, OMC and Ti_3_C_2_T_*x*_/OMC (prepared by simply mixing the same proportion of Ti_3_C_2_T_*x*_ and OMC as in the Ti_3_C_2_T_*x*_-OMC) are also shown. The cyclic voltammetry (CV) curves of the Ti_3_C_2_T_*x*_-OMC sample exhibit a higher current, and thus, a larger capacitance, than that of the Ti_3_C_2_T_*x*_ and Ti_3_C_2_T_*x*_/OMC samples (Fig. 4a and Supplementary Fig. 8). Based on the galvanostatic charge/discharge (GCD) curves shown in Supplementary Fig. 9, the gravimetric capacitance are calculated and plotted in Fig. 4b. The gravimetric capacitances of the Ti_3_C_2_T_*x*_-OMC and Ti_3_C_2_T_*x*_/OMC mixture at 1 A g^−1^ are 64 and 55 F g^−1^, respectively, whereas the capacitance of Ti_3_C_2_T_*x*_ is only 52 F g^−1^ (Fig. 4b). The superior capacitance of Ti_3_C_2_T_*x*_-OMC is attributed to the insertion of the OMC, which can increase the surface area, introduce electric double layer capacitance and enhance the ion-accessible trap sites, thus facilitating the ion intercalation. Upon gradually increasing the current density, the GCD profiles of the Ti_3_C_2_T_*x*_-OMC remain symmetrical (Supplementary Fig. 9a) and a capacitance as high as 53 F g^−1^ can be retained at 10 A g^−1^. This value is much higher than that of the Ti_3_C_2_T_*x*_ electrode (38 F g^−1^) at the same current density, highlighting the faster ion-diffusion and high-charge storage behaviour (Fig. 4b). The volumetric capacitance of all the electrodes was also calculated. Especially, albeit the Ti_3_C_2_T_*x*_-OMC has a slightly lower density than Ti_3_C_2_T_*x*_ (Supplementary Table 1), the volumetric capacitance of Ti_3_C_2_T_*x*_-OMC still reaches 198 F cm^−3^ (Fig. 4c) at a current density of 1 A g^−1^, which is superior to that of pure Ti_3_C_2_T_*x*_ electrode (177 F cm^−3^) in this work. The Nyquist plots of Ti_3_C_2_T_*x*_-OMC and Ti_3_C_2_T_*x*_ are displayed in Fig. 4d. The high-frequency intercept on the Z′ axis represents the total resistances which combines the intrinsic resistance of active material, the contact resistance between the active material and the current collector, and the electrolyte resistivity^32^. Therefore the high-frequency resistance of the Ti_3_C_2_T_*x*_-OMC is slightly smaller than that of Ti_3_C_2_T_*x*_ in spite of the higher electrical conductivity of Ti_3_C_2_T_*x*_-OMC (explained in detail in Supplementary Table 1 and Supplementary Note 3). The shorter Warburg district in middle-frequency region suggests that the ion diffusion efficiency in the Ti_3_C_2_T_*x*_-OMC electrode is higher.

**Figure 5:**
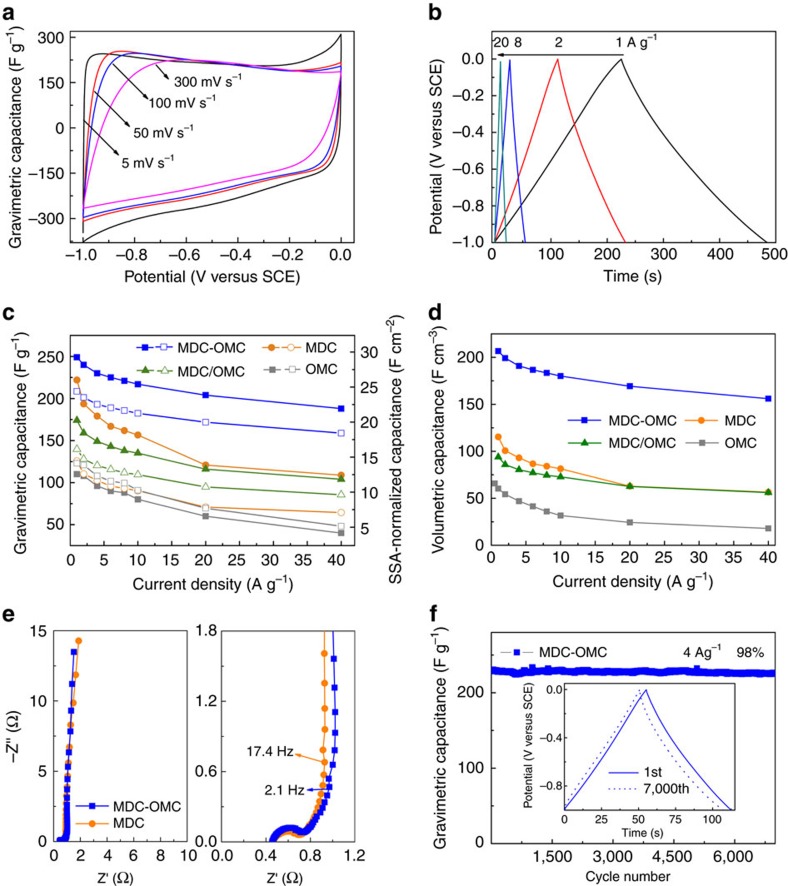
Electrochemical performance of MDC-OMC. (**a**) CV curves of the MDC-OMC at various scan rates. (**b**) GCD curves of the MDC-OMC at different current densities. (**c**) Comparison of gravimetric capacitances (the solid symbols) and SSA-normalized capacitances (the hollow symbols) and (**d**) volumetric capacitances versus different current densities for the MDC-OMC, MDC/OMC, MDC and OMC. (**e**) Nyquist plots of the MDC-OMC and MDC with the close-up view on the high-frequency regime. (**f**) Cycling performance of the MDC-OMC. Inset: The corresponding galvanostatic charge/discharge curves at a current density of 4 A g^−1^ of 1st and 7,000th cycle. The reference electrode is a SCE.

After high temperature chlorination, Ti was completely etched from Ti_3_C_2_T_*x*_-OMC and Ti_3_C_2_T_*x*_ to produce the respective pure carbon materials of MDC-OMC and MDC with high SSA. It is well understood that carbon materials with high SSA and accessible pores are ideal candidates as electrode for electric double layer capacitor (EDLC), in which the energy storage is based on the ion adsorption on the electrode surface. The nearly rectangular CV curves and symmetrical GCD profiles of the MDC-OMC (Fig. 5a,b) are ideal features for an efficient EDLC. The MDC-OMC electrode shows much larger CV curve areas and longer discharge time (higher gravimetric capacitance) compared to the other electrodes (Supplementary Fig. 10). As plotted in Fig. 5c, the specific capacitance of MDC-OMC (249 F g^−1^) at a current rate of 1 A g^−1^ is higher than those of MDC (222 F g^−1^), MDC/OMC (179 F g^−1^) and OMC (110 F g^−1^) and that of most reported carbon electrode (50–200 F g^−1^, Supplementary Table 2)^33^,^34^,^35^,^36^,^37^. After increasing the current density, the MDC-OMC continues to provide well-behaving GCD curves and high capacitances, achieving 188 F g^−1^ at 40 A g^−1^, which is much higher than other electrodes. It is expected that integrating mesopores inside a microporous structure can not only improve the ion-accessible surface area, but also facilitate fast electrolyte movement through the carbon matrix. What is of particular interest is that a high capacitance is achieved for the MDC-OMC, although it possesses a much lower surface area than other typical carbon-based electrodes (1,500–2,000 m^2^ g^−1^) (ref. 37). The surface area-normalized capacitance at 1Ag^−1^ for the MDC-OMC is much higher (24 μF cm^−2^) than those for the MDC (15 μF cm^−2^), MDC/OMC (17 μF cm^−2^), OMC (14 μF cm^−2^) and activated carbon materials (5∼22 μF cm^−2^) (Fig. 5c)^10^,^38^,^39^. Moreover, the bulk density of MDC-OMC electrode is about 0.83 g cm^−3^, which is much higher than that of the MDC (0.52 g cm^−3^) or traditional activated carbon (0.5-0.7 g cm^−3^) (as explained in detail in Supplementary Table 1 and Supplementary Note 4)^40^. Therefore, the corresponding volumetric capacitance of the MDC-OMC electrode achieves 212 F cm^−3^ at 0.5 A g^−1^ (Fig. 5d), which is better than or comparable to the best carbon materials previously reported (Supplementary Table 2). All these results verify that the MDC-OMC provide full access to the electrolyte without geometrical or electrical hindrance, even with high packing density. In future works, the capacitance could be further enhanced by doping heteroatoms, such as nitrogen, boron and phosphorus, to introduce pseudocapacitance^41^,^42^,^43^. As shown in the Nyquist plots (Fig. 5e), the semi-circle in the high-frequency region is described as an intrinsic electron-transfer resistance in the electrode materials. For EDLC, it is associated with the porous structure of the carbon electrode^32^,^44^. Since the high-frequency resistances are influenced by many factors, the semi-circle of MDC-OMC and MDC are almost the same in spite of the different intrinsic resistance (explained in detail in Supplementary Table 1 and Supplementary Note 3). The Nyquist plots of the MDC-OMC are steeper in the low-frequency region (Fig. 5e, left) and exhibit shorter resistance-capacitance transmission in the middle-frequency region (Fig. 5e, right) than the MDC, indicating that the capacitance of the MDC-OMC can be fully reached at a faster charging rate than MDC. After increasing the areal density of the electrode film from 5 to 12 mg cm^−2^ (the thickness of electrode film increases from 60 to 120 μm), the MDC-OMC electrode maintains its EDLC behaviour. The capacitance only decreases slightly from 249 to 240 F g^−1^ at a current density of 1 A g^−1^ (Supplementary Fig. 11a,b) and the equivalent series resistance slightly increased from 0.74 Ω (Fig. 5e) to 0.8 Ω (Supplementary Fig. 11c). Therefore, more energy and power is expected to be stored in the MDC-OMC-based cell when more active electrode materials are assembled. Moreover, the MDC-OMC exhibits a remarkable cycling stability, with a capacitance retention of more than 98% after 7,000 cycles at a current density of 4 A g^−1^ (Fig. 5f), which is critical for practical applications.

To further evaluate the effect of the 2D–2D heterostructure on the ion conductivity, we have implemented the MDC-OMC in a symmetrical two-electrode system in 1 M tetraethylammonium tetrafluoroborate (TEA BF_4_) in acetonitrile (AN) to evaluate the supercapacitor performance. Each CV curve (from 10 to 1,000 mV s^−1^) displays a typical rectangular shape between 0 and 2.5 V, suggesting that the MDC-OMC in organic electrolytes have pure electric double layer capacitive properties (Supplementary Fig. 12a). The discharging plots are generally a symmetric reflexion of their corresponding charging counterparts, revealing high-capacitive reversibility of the MDC-OMC (Supplementary Fig. 12b). The specific capacitance calculated based on the discharge curve at a current density of 1 A g^−1^ was 162 F g^−1^. This value is higher than those of carbide-derived carbon and some porous carbon-based electrode materials (Supplementary Table 2). When the current density is increased up to 4, 20 and 60 A g^−1^, the specific capacitance of the MDC-OMC decreases to 159, 144, 132 F g^−1^, respectively (Supplementary Fig. 12c). Supplementary Fig. 12d shows a Nyquist plot of MDC-OMC over the frequency range of 10^−2^ to 10^5^ Hz and a close-up view of the high-frequency region in the inset. The Nyquist plots show the complex-plane (Nyquist) impedance for both electrodes, which is an indication of the capacitive characteristics of a test device (imaginary component, Z″) versus the Ohmic impedance (real component, Z′). The Nyquist plot for porous electrodes is typically divided into two regions by the knee frequency: the critical frequency at which all surface area is accessed, that is, saturated^45^,^46^. The knee frequency of the MDC-OMC and MDC-based EDLC are 90.2 and 72.1 Hz, respectively. Supplementary Fig. 12e shows the dependence of phase angle on the frequency for MDC-OMC and MDC. The MDC-OMC exhibits a frequency of 1.96 Hz at a phase angle of 45°, corresponding to a time constant of 0.51 s, which is much shorter than for the MDC (2.32 s). The higher knee frequencies and shorter time constant induced by the good accessibility of the ions into the 2D–2D heterostructure strongly demonstrate the excellent power capability of the MDC-OMC. These results support that the 2D–2D heterostructure, directed mesopores and large surface area participate to enable electrolyte penetration throughout the material, leading to a high capacitance and rate capability. As shown in the Ragone plot (Supplementary Fig. 12f), the maximum energy density of MDC-OMC supercapacitor achieves 35.6 Wh kg^−1^ at a power density of 0.63 kW kg^−1^. At the maximum power density of 196.5 kW kg^−1^, the energy density remains as high as 30.6 Wh kg^−1^.

## Discussion

This work proposed to prepare 2D–2D heterostructured composites by assembling 2D Ti_3_C_2_T_*x*_ (or Ti_3_C_2_T_*x*_-derived MDC) nanosheets and 2D OMC layers. The key points of this strategy are to prepare 2D nanosheets host with abundant hydrophilic surface termination, and control the molecular weight of guest organic molecules and block copolymers to allow their easy intercalation within the narrow interlayer space in order to confine the close-packing assembly of block copolymer micelles. The thickness of the intercalated OMC layer is dependent on the interlayer distance of the 2D host due to the confinement effect. When used as electrode for supercapacitor, the Ti_3_C_2_T_*x*_-OMC and MDC-OMC samples exhibit superior performance than the pristine 2D materials. The improved capacitive performance should be attributed to the synergetic effects of the 2D–2D heterostructure. The interconnected structures consisting of 2D nanosheets and mesopores allow easier ion transport to reach the electroactive sites in the bulk materials and enhance the utilizations of electrodes, giving rise to an increased capacitance. The aligned mesopores can also serve as an electrolyte reservoir, which significantly shorten the diffusion paths and improve the transport efficiency. In addition, the mesoporous carbon intercalated in the layers provides a continuous electron pathway between two adjacent MDC sheets (*c*-direction), ensuring good electrical conductivity.

In summary, this work paves a pathway for solving the stacking and connection problem in 2D nanosheets by constructing regular, vertical and accessible porous pillars within the 2D interlayers. This versatile approach can be expected to be further adapted for the direct patterning of mesoporous carbon on the surface of 2D materials. From the view of practical application, the concept proposed here, nanofabrication of vertical accessible pillars inside 2D layers, will open a new paradigm for the synthesis of 2D hybrid nanomaterials to target a broad variety of applications. In further research, this strategy can be extended to prepare other 2D–2D hybrid materials.

## Methods

### Synthesis of Ti_3_C_2_T_*x*_

Roughly 10 g of Ti_3_AlC_2_ MAX phase was immersed in 100 ml of a 45% concentrated HF solution (Wako) at room temperature for 6 h to extract Al. The resulting suspension was then washed several times using deionized water and centrifuged to isolate the Ti_3_C_2_T_*x*_ powders with multilayers. To obtain few-layer Ti_3_C_2_T_*x*_, multilayered Ti_3_C_2_T_*x*_ was immersed in dimethyl sulfoxide for 18 h at room temperature, and then centrifuged to separate the intercalated Ti_3_C_2_T_*x*_ powder. Deionized water was added with a weight ratio of Ti_3_C_2_T_*x*_ to water of 1:500. Then the suspension was ultrasonicated under argon for 4 h and centrifuged for 1 h at 3,500 r.p.m. After filtering the decanted supernatant and drying under vacuum, the few-layer Ti_3_C_2_T_*x*_ was obtained. Finally, 0.1 g few-layer Ti_3_C_2_T_*x*_ was dispersed in 100 ml ethanol for further synthesis.

### Synthesis of resol and F127 unimers suspension

Low-molecular-weight phenol-formaldehyde resol was first synthesized according to an established method^47^. In a typical procedure, 0.61 g of phenol was melted at 40–42 °C and mixed with 0.13 of NaOH aqueous solution (20 wt%). After stirring for 10 min, 1.05 g of formaldehyde solution (37 wt%) was added dropwise. Upon further stirring at ∼75 °C for 1 h, the solution was cooled to room temperature and the pH value was adjusted to ∼7.0 with HCl solution. After removing water by vacuum evaporation below 50 °C, the resol was obtained. Then 0.1 g resol and 0.1 g triblock copolymer Pluronic F127 (Mw=12,600, PEO106-PPO70-PEO106, Aldrich.) were dispersed in 100 ml ethanol solution, respectively.

### Synthesis of Ti_3_C_2_T_*x*_-OMC composite

The above pre-prepared ethanolic solution of Ti_3_C_2_T_*x*_ and resol-F127 unimers were mixed and stirred for at least 6 h at room temperature. The obtained homogeneous solution was transferred to a flat dish and dried under vacuum until the ethanol completely evaporated out, followed by aging at 100 °C for 24 h. Afterward, the Ti_3_C_2_T_*x*_-OMC sample was obtained by carbonization at 900 °C in nitrogen for 2 h.

### Synthesis of MDC-OMC composite and MDC

The MDC-OMC and MDC samples were obtained by treating Ti_3_C_2_T_*x*_-OMC and Ti_3_C_2_T_*x*_, respectively, in chlorine gas at 900 °C for 2 h. Then the sample was treated in ammonia at 600 °C for 2 h to remove residual chlorine from the highly porous material.

### Synthesis of OMC

The above pre-prepared ethanolic solution of resol-F127 unimers was transferred to a flat dish and dried under vacuum until the ethanol completely evaporated out, followed by aging at 100 °C for 24 h. Afterward, the OMC sample was obtained by carbonization at 900 °C in nitrogen for 2 h.

### Characterization

The morphology of the samples was observed using a Hitachi SU-8000 field-emission SEM. The TEM observation was conducted at 200 kV on a JEOL JEM-2100 equipped with energy-dispersive X-ray spectroscopic analysis. Powder XRD analysis was operated on Rigaku Rint 2000 X-ray 27 diffractometer with monochromated Cu Kα radiation. Raman spectroscopy was carried out on a HORIBA Scientific Lab RAM HR Raman spectrometer system using a 532.4 nm laser. TG analysis was performed on a Hitachi HT-Seiko Instrument Exter 6300 TG/DTA 9 in Air heating from room temperature to 900 °C (5 °C min^−1^). The nitrogen adsorption–desorption isotherms of the samples were acquired by using a Micromeritics BK122T–B analyzer. The SSA was determined according to Brunauer–Emmett–Teller theory in the relative pressure range of 0.04 to 0.2. Pore size distributions were determined from the adsorption branches of the isotherms, based on the NLDFT. The electrical conductivity was determined by pressing the samples between two plungers into a hollow Nylon cylinder, and applying a pressure of up to 12.5 MPa.

### Electrochemical measurement

Typically, the electrode was prepared by mixing the active material (85 wt%), acetylene black (10 wt%) and poly(tetrafluoroethylene) (5 wt%). The electrode was rolled into a film and dried under vacuum at 100 °C for 12 h. The film was cut into tablets of ∼1 cm^2^ and then pressed onto nickel foam or aluminium foil as working electrode for three-electrode system or symmetric supercapacitor, respectively. All the electrochemical measurements were carried out on a CHI 660D electrochemical workstation. The electrochemical impedance spectroscopy measurements were performed at open circuit potential in the frequency range of 10^–2^ to 10^5^ Hz at an AC amplitude of 5 mV. The cycle life tests were conducted by GCD measurements at 4 A g^–1^. In the three-electrode system, a platinum plate electrode and a saturated calomel electrode served as counter and reference electrodes, respectively. The two electrodes separated by glass fibre film were soaked into 1 M tetraethylammonium tetrafluoroborate (TEA BF_4_) in acetonitrile (AN) and assembled into a coin-type cell. The density of the electrode film was calculated according to: 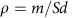, where *m* is the mass, *S* is the area and *d* is the thickness of electrode film. For the three-electrode system, the gravimetric capacitance (*C*_m_) of electrode was calculated from the galvanostatic discharge curve according to: 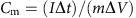, where *I* is current, Δ*t* is discharge time, *m* is the mass of active material and Δ*V* is the voltage range. The volumetric capacitance (*C*_V_) of electrode was calculated using: 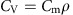. For the two-electrode device, the gravimetric capacitance (*C*_M_) of one electrode was calculated from the galvanostatic discharge curve according to: 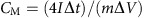, where *I* is current, Δ*t* is discharge time, *m* is the total mass of active material and Δ*V* is the voltage range. The energy density against the two-electrode device was calculated according to: 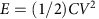, where *C* is the capacitance of device and *V* is voltage applied. The power density was calculated according to: 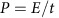, where *t* is the discharge time.

### Data availability

The data that support the findings of this study are available within the paper and its supplementary information file, or from the corresponding author on request.

## Additional information

**How to cite this article:** Wang, J. *et al*. Hierarchical porous carbons with layer-by-layer motif architectures from confined soft-template self-assembly in layered materials. *Nat. Commun.* **8**, 15717 doi: 10.1038/ncomms15717 (2017).

**Publisher's note:** Springer Nature remains neutral with regard to jurisdictional claims in published maps and institutional affiliations.

## Supplementary Material

Supplementary Information Supplementary Figures, Supplementary Tables, Supplementary Notes and Supplementary References
