# Supplementary material for: Hierarchical porous carbons with layer-by-layer motif architectures from confined soft-template self-assembly in layered materials
Source: Nat Commun. 2017 Jun 12;8:15717. doi: 10.1038/ncomms15717 (PMC5472787; doi:10.1038/ncomms15717)
Supplement: Supplementary Information — Supplementary Figures, Supplementary Tables, Supplementary Notes and Supplementary References [file ncomms15717-s1.pdf]

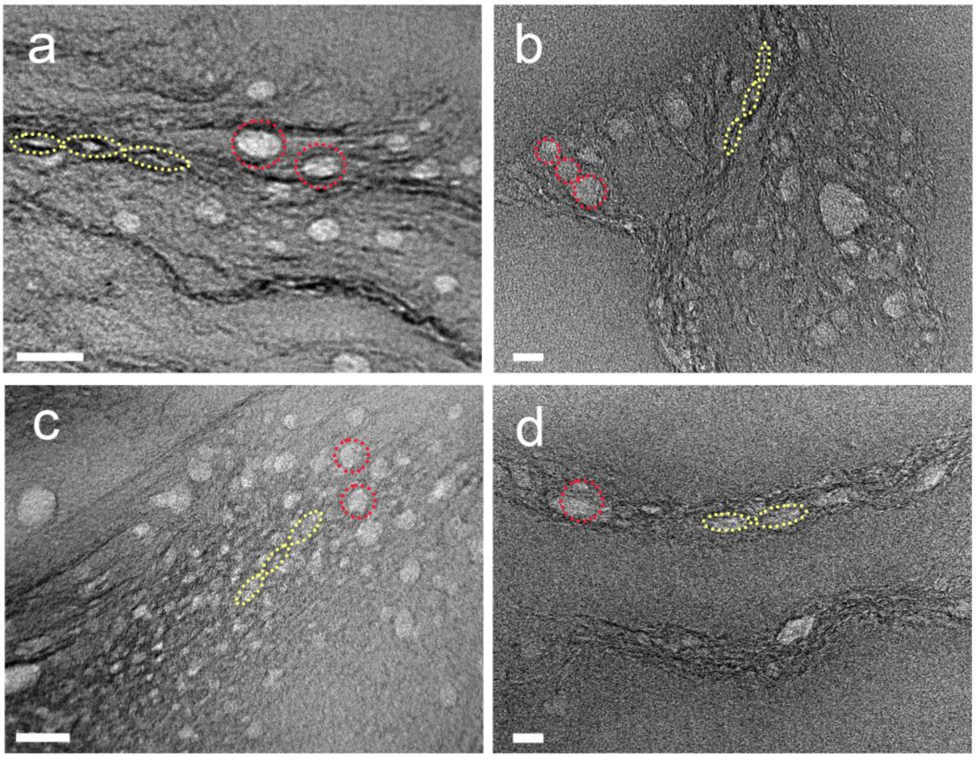

Supplementary Figure 1. (a-d) Cross-section TEM images of the MDC-OMC. Scale bars are 50 nm for a, c and 20 nm for b, d.

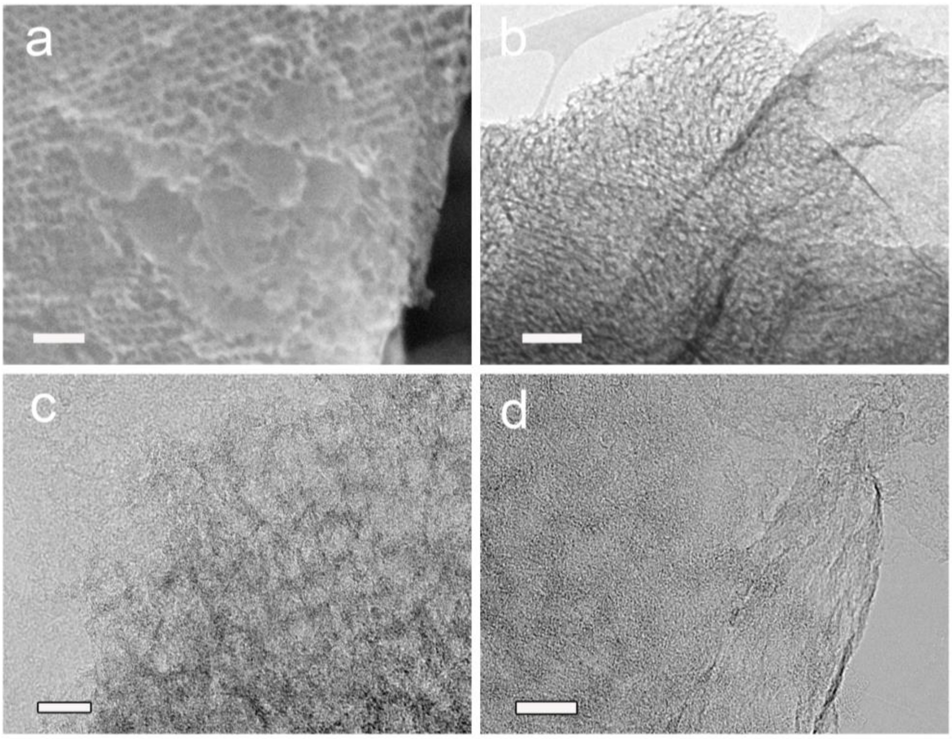

Supplementary Figure 2. (a) Top-view SEM and (b-d) TEM images of the MDC-OMC. Scale bars are 50 nm for a, 100 nm for b and 20 nm for c, d.

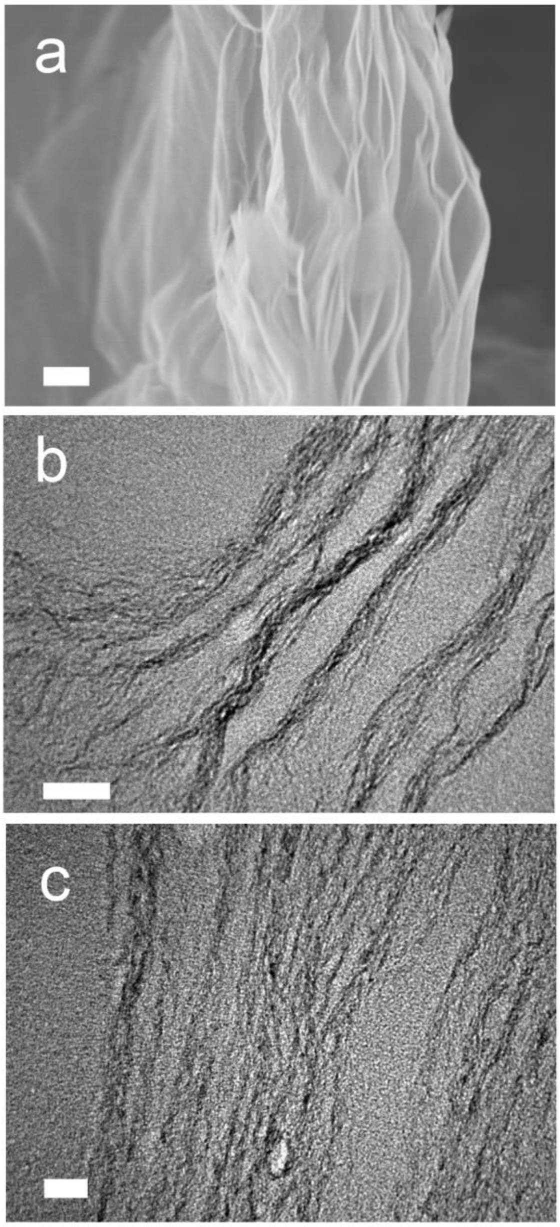

Supplementary Figure 3. (a) Cross-section SEM image of the MDC. (b-c) Cross-section TEM images of MDC. Scale bars are 100 nm for a, 50 nm for b and 20 nm for c.

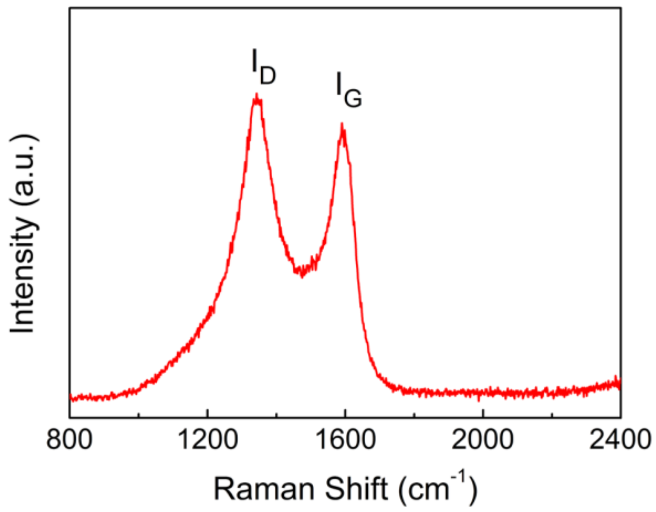

Supplementary Figure 4. Raman spectrum of the MDC-OMC.

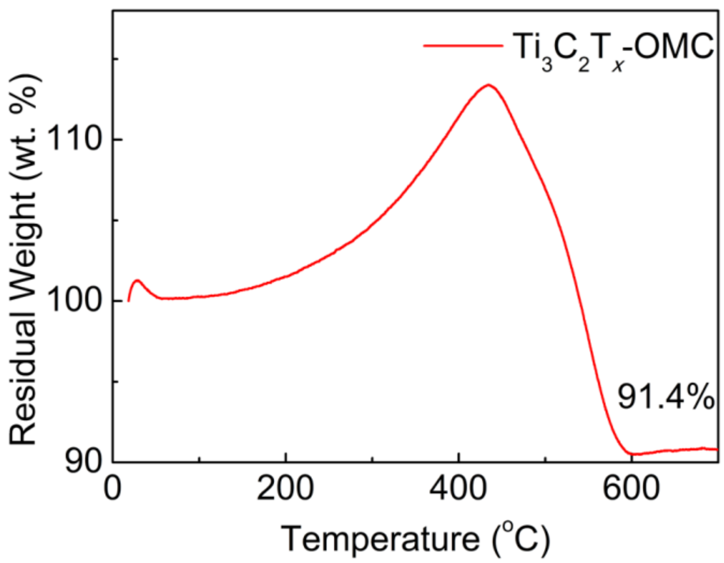

Supplementary Figure 5. TG curve of the $Ti_3C_2T_x$-OMC in air.

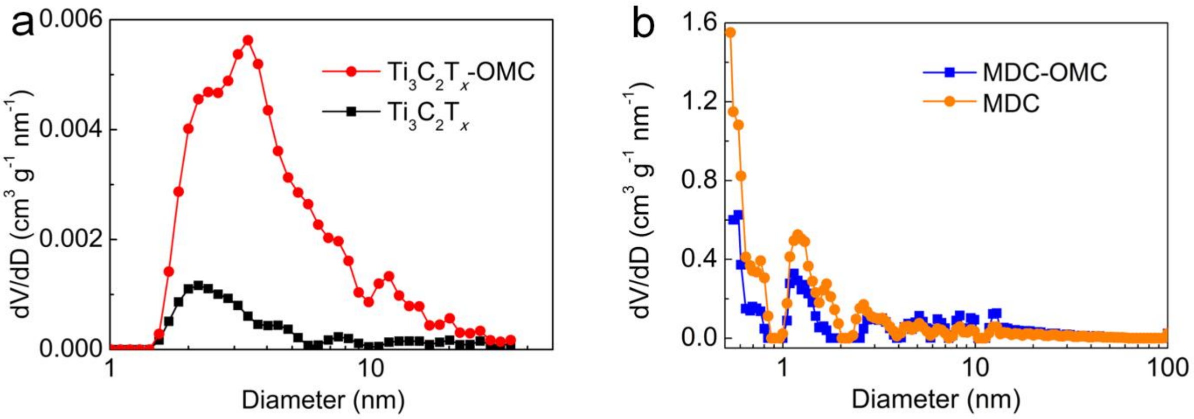

Supplementary Figure 6. Pore size distribution curves of (a) the $Ti_3C_2T_x$ and $Ti_3C_2T_x$-OMC and (b) the MDC and MDC-OMC.

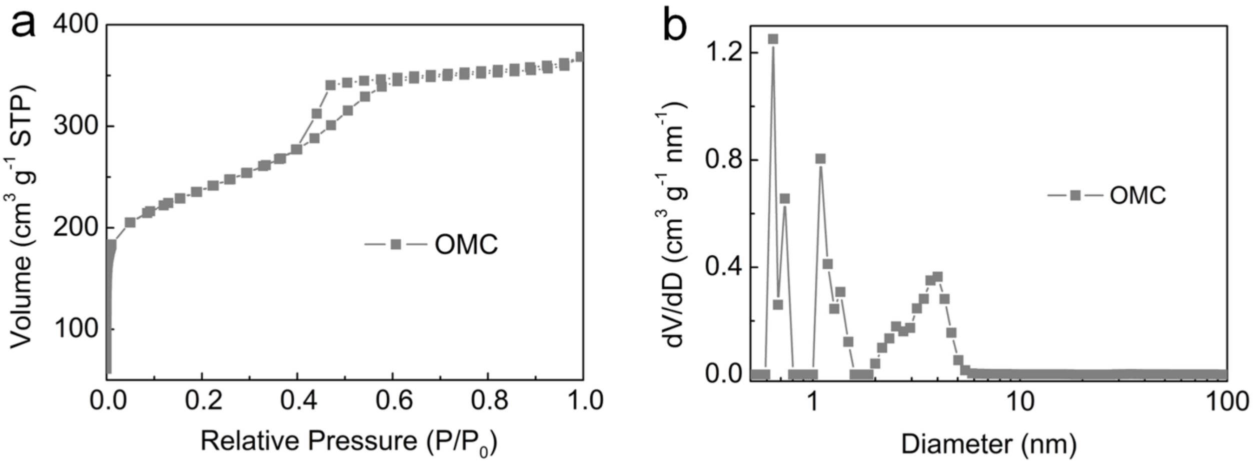

Supplementary Figure 7. (a) Nitrogen adsorption-desorption isotherms of the OMC and (b) corresponding pore size distribution curve.

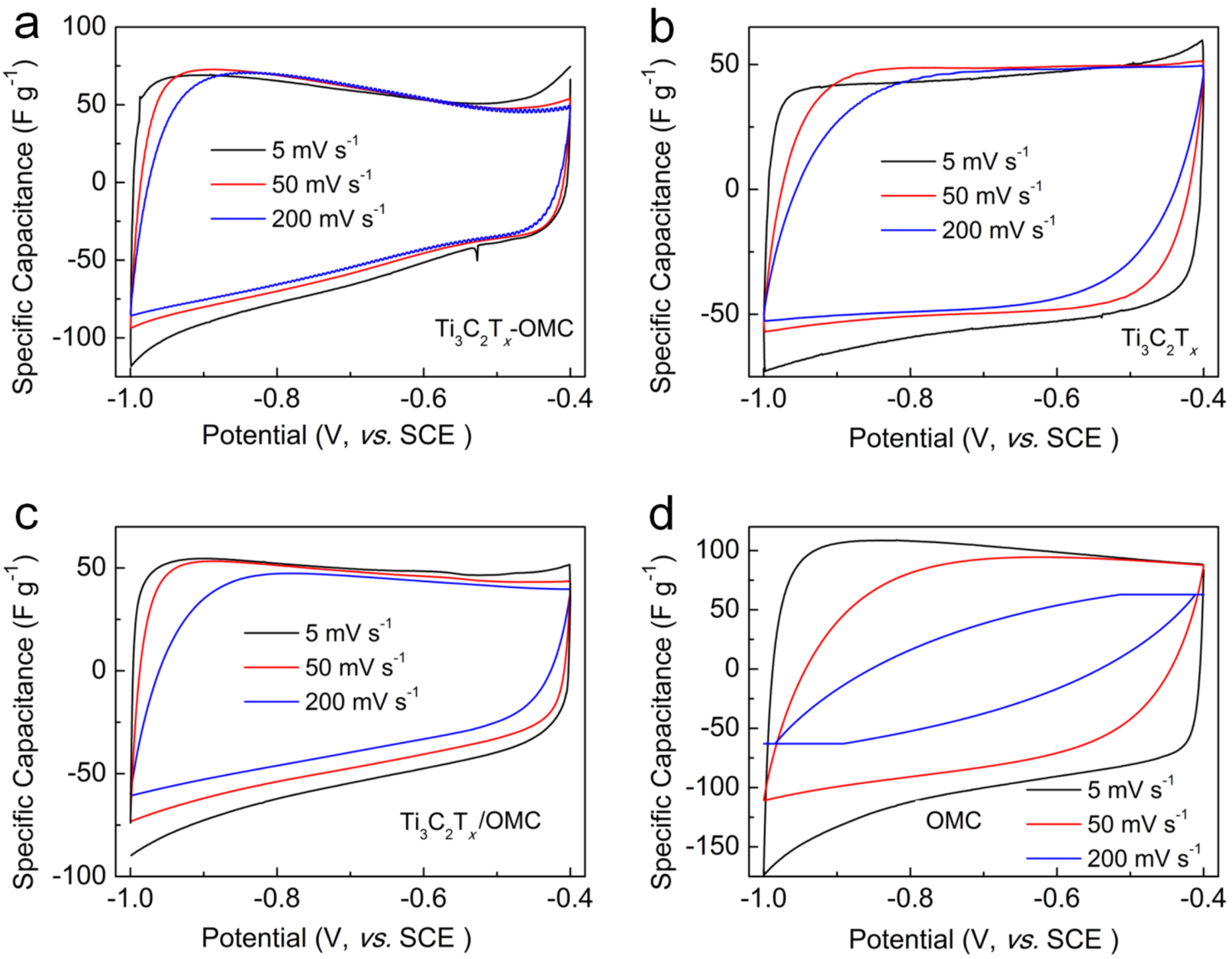

Supplementary Figure 8. CV curves of (a) the $Ti_3C_2T_x$-OMC, (b) $Ti_3C_2T_x$, (c) $Ti_3C_2T_x$/OMC mixture, and (d) OMC at various scan rates in 6 M KOH electrolyte using a three-electrode system.

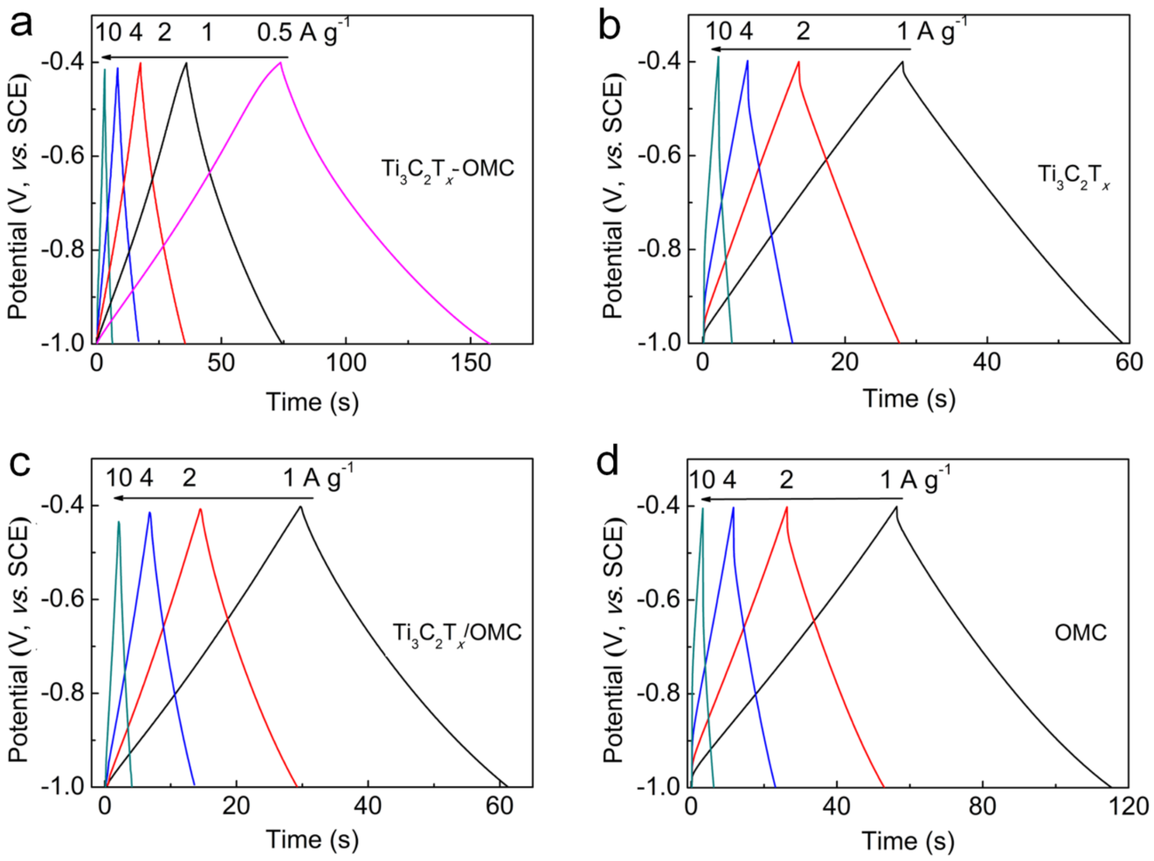

Supplementary Figure 9. GCD curves of (a) the $Ti_3C_2T_x$-OMC, (b) $Ti_3C_2T_x$, (c) $Ti_3C_2T_x$/OMC mixture, and (d) OMC at various current densities in 6 M KOH electrolyte using a three-electrode system.

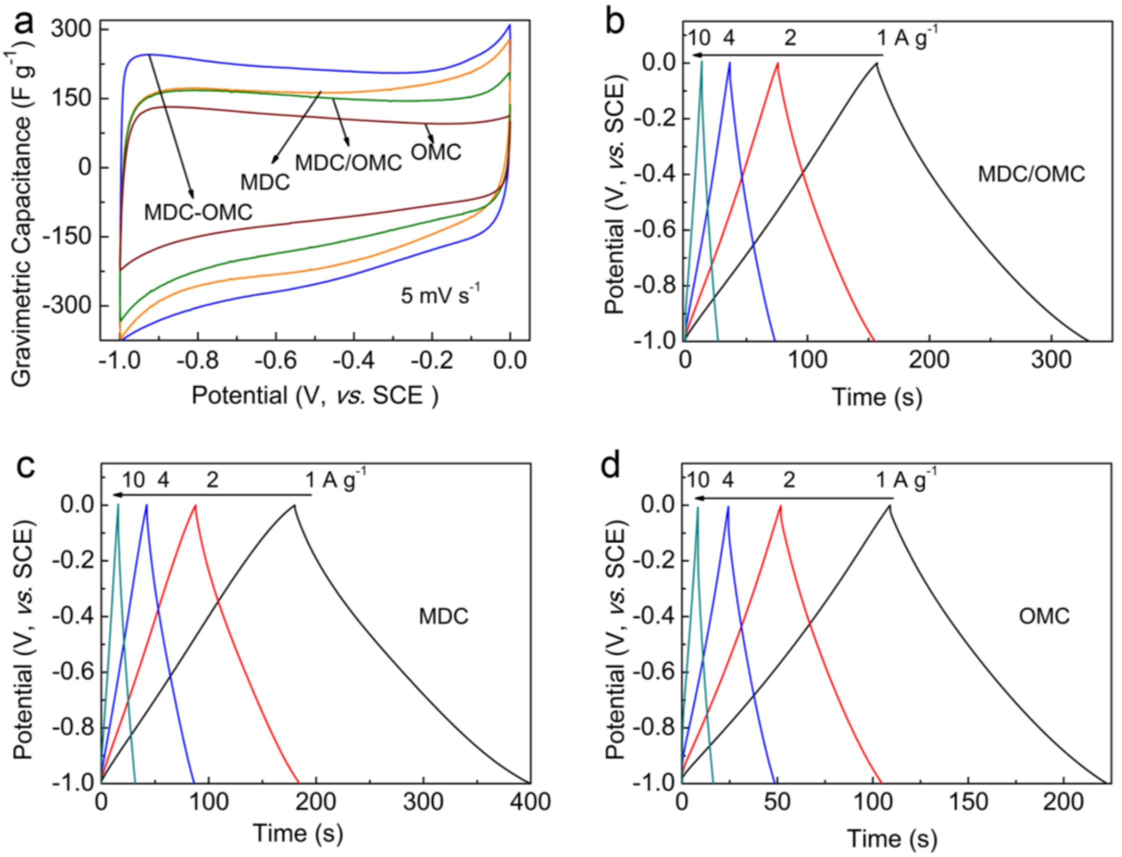

Supplementary Figure 10. (a) CV curves of the MDC-OMC, MDC/OMC mixture, MDC, and OMC at a scan rate of 5 mV s^{-1}. GCD curves of (b) the MDC/OMC mixture, (c) MDC, and (d) OMC at various current densities in 6 M KOH electrolyte using a three-electrode system.

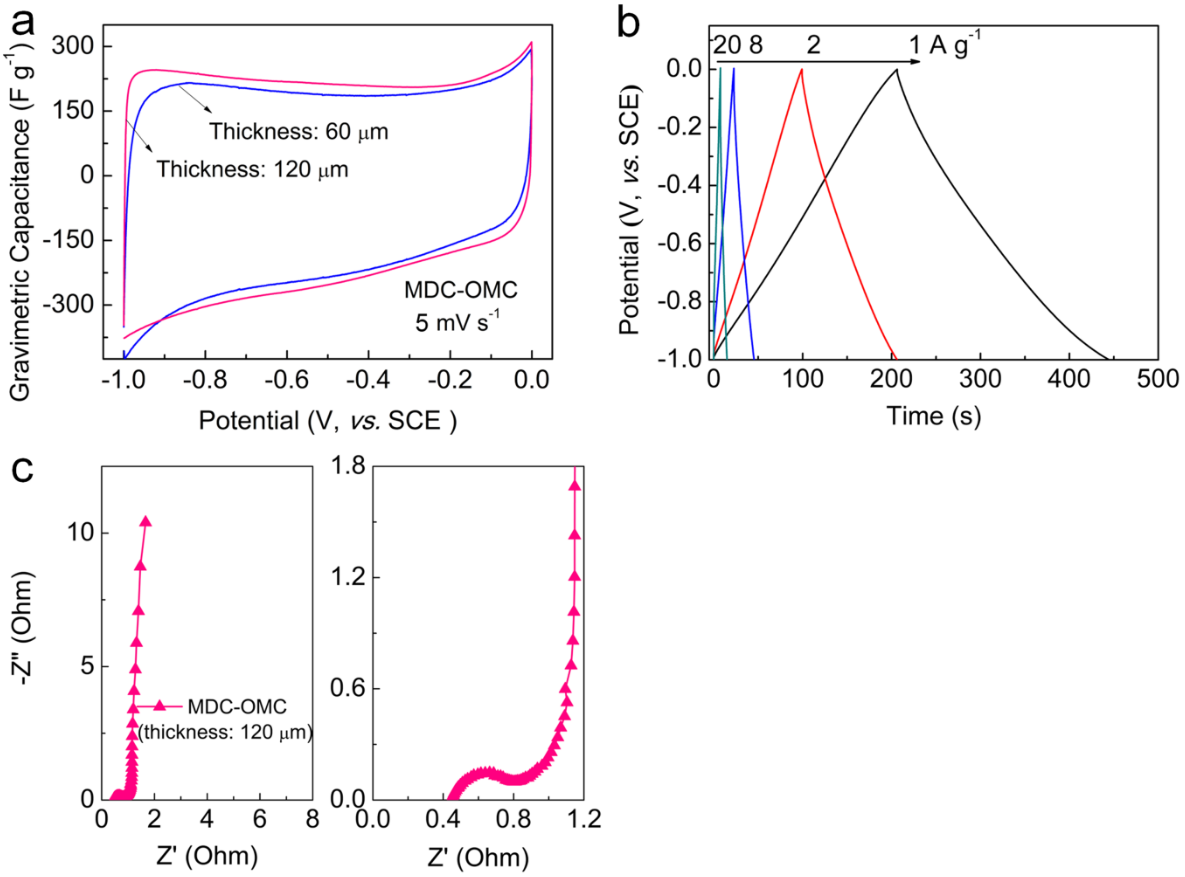

Supplementary Figure 11. (a) CV curves of the MDC-OMC electrodes with different thicknesses. (b) GCD curves of the MDC-OMC electrode with a thickness of 120 μm. (c) Nyquist plots of the MDC-OMC electrode of 120 μm thickness with the close-up view of the high-frequency regime.

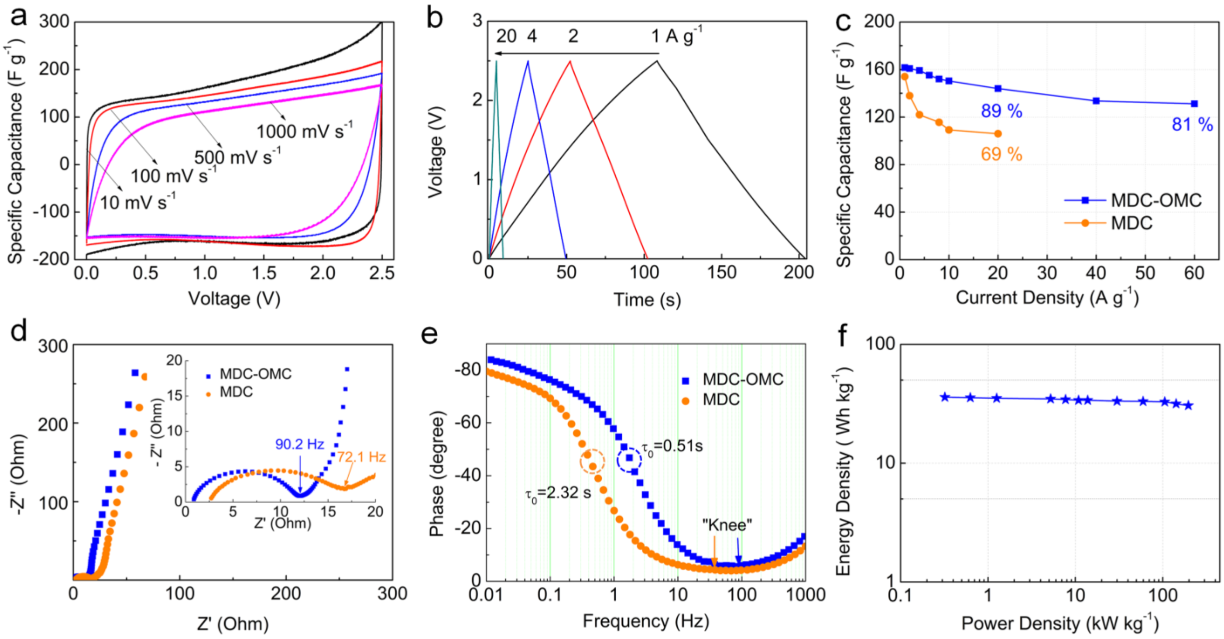

Supplementary Figure 12. Electrochemical performance of the MDC-OMC and MDC in 1 M TEA BF_4 in AN. (a) CV curves of the MDC-OMC at scan rates of 10, 100, 500 and 1000 mV s^{-1}. (b) Galvanostatic charge/discharge curves of the MDC-OMC at current densities of 1, 2, 4 and 20 A g^{-1}. (c) Comparison of specific capacitances *vs.* various current densities for the MDC-OMC and MDC. (d) Nyquist plots of the MDC-OMC and MDC. Inset magnifies the high-frequency regime. (e) Bode plots of phase angle *vs.* frequency for the MDC-OMC and MDC. (f) Ragone plots showing energy density and power density of the MDC-OMC supercapacitor.

Supplementary Table 1. Summary of the specific surface area (SSA), pore volume (PV), electrical conductivity for $Ti_3C_2T_x$-OMC, $Ti_3C_2T_x$/OMC mixture, $Ti_3C_2T_x$, MDC, OMC, MDC-OMC, and MDC/OMC mixture samples. The densities of corresponding electrodes are also reported.

Sample	BET SSA/ m^2 g^{-1}	Micro-SSA/ m^2 g^{-1}	Ratio of Micro-SSA/BET SSA	Total PV/ cm^3 g^{-1}	Micro-PV/ cm^3 g^{-1}	Ratio of micro-PV/Total PV	Conductivity/ S cm^{-1}	Density /g cm^{-3}
$Ti_3C_2T_x$	13	1.1	9%	0.05	0	0%	168	3.4
$Ti_3C_2T_x$-OMC	84	18	22%	0.19	0.02	11%	187	3.1
$Ti_3C_2T_x$/OMC	92	43	47%	0.05	0.035	70%	124	2.4
MDC	1536	894	58%	1.23	0.39	32%	67	0.52
OMC	779	379	49%	0.57	0.28	49%	8	0.61
MDC-OMC	1021	367	36%	1.62	0.16	10%	81	0.83
MDC/OMC	1080	600	56%	0.80	0.42	53%	44	0.54

Supplementary Table 2. Summary of the supercapacitive performance of representative porous carbon electrodes.

Name of typical material	Scan rate/ Current density	C_g/ F g^{-1}	C_V/ F cm^{-3}	Electrolyte	Ref
Commercial activated carbon	-	160-200	80-110	6M KOH	1
Charcoal-derived porous carbon	1 A g^{-1}<br>10 A g^{-1}	186<br>168 (90%)	106<br>96	1M TEA BF_4/AN	2
MOF-derived carbon nanorods	0.05 A g^{-1}	198	-	1M H_2SO_4	3
Porous carbon nanosheets	0.5 A g^{-1}	257	-	6M KOH	4
Porous carbon film	10 mV s^{-1}	240	220	0.5M H_2SO_4	5
Reduced graphene oxide (RGO) paper	0.5 A g^{-1}	208	196	6M KOH	6
EM-CCG film	0.1 A g^{-1}	191.7	255.5	1M H_2SO_4	7
Compact RGO film	5 mV s^{-1}	185	226	6M KOH	8
Graphene/OMC	0.5 A g^{-1}	197	-	6M KOH	9
Graphene/CNT films	50 mV s^{-1}	175	160	0.5M H_2SO_4	10
Graphene oxide and nanodiamond	0.2 A g^{-1}	143	-	1M H_2SO_4	11
Carbide derived carbon (CDC) film	20 mV s^{-1}	-	160	1M H_2SO_4	12
Graphene-like CDC	20 mV s^{-1}	80	-	1.5 M TEA BF_4/AN	13
MDC	1 A g^{-1}	222	-	6M KOH	14
MAX derived carbon	0.5 A g^{-1}	160	-	6M KOH	14
$Ti_3C_2T_x$-OMC	0.5 A g^{-1}	67	208	6M KOH	This

	5 mV s^{-1}	65	202		work
MDC-OMC	0.5 A g^{-1}	255	212	6M KOH	This work
	10 A g^{-1}	217 (85%)	180		
	5 mV s^{-1}	234	194		
	500 mV s^{-1}	203 (87%)	169		
MDC-OMC	1 A g^{-1}	162	135	1M TEA BF_4/AN	This work
	10 A g^{-1}	151 (93%)	125		

Supplementary Note 1. Detailed discussion on Raman spectrum.

There are two distinct vibration bands shown in the Raman spectrum of MDC-OMC (**Supplementary Fig. 4**). The D band (1360 cm^{-1}) generally originates from the vibrations of disordered carbon or the defects in the plane terminations, whereas the G band (1590 cm^{-1}) is related to the vibrations of ordered sp^2-bonded graphitic carbon sheets. Raman spectrum of MDC-OMC implies the coexistence of disordered carbon and ordered graphitic carbon.

Supplementary Note 2. Detailed discussion on ICP and TG analysis.

As determined by the ICP analysis, the weight percentage of Ti and C in the $Ti_3C_2T_x$-OMC is about 56% and 24.5±0.1%, respectively. It can be reckoned that the weight percentage of $Ti_3C_2T_x$ is ~85% and OMC is ~15%. After chlorination, the percentage of MDC and OMC in the MDC-OMC is about 38.4% and 61.6%, respectively.

We also carried out thermogravimetric analysis (TG) in air to estimate the weight percentage of Ti and C in the $Ti_3C_2T_x$-OMC. As shown in **Supplementary Fig. 5**, the Ti starts to be oxidized at ~100 °C with a continuous weight increase until ~435 °C. When the temperature further increased higher than 435 °C, the weight reduced dramatically because of the decomposition of carbon in air. The carbon was burnt out until 600 °C and the remaining weight appeared stable. The remaining is TiO_2 with a weight percentage of 91.4%. It can be reckoned that the percentage of Ti in the original $Ti_3C_2T_x$-OMC is ~55%. The reduced weight percentage above 400 °C corresponds to the content of carbon, which is ~22.8%. These results are consistent with the ICP analysis.

Supplementary Note 3. Detailed discussion on electrical conductivity of $Ti_3C_2T_x$, MDC, and MDC-OMC.

The electrical conductivity of $Ti_3C_2T_x$ (168 S cm^{-1}) in this work is lower than the previously reported $Ti_3C_2T_x$ film (2.4×10^3 S cm^{-1}) [15]. This could be attributed to the following two effects: (1) The $Ti_3C_2T_x$ film with high electrical conductivity was prepared from the exfoliated $Ti_3C_2T_x$ nanosheets through vacuum filtration. Thus the tightly packed and continuously connected $Ti_3C_2T_x$ nanosheets would possess better electron transfer properties; (2) The electrical conductivity of the $Ti_3C_2T_x$ powder is highly dependent on the particle sizes due to the particle-to-particle contact resistance, thus, $Ti_3C_2T_x$ materials composed of small blocks would have a lower electrical conductivity compared to the large-sized $Ti_3C_2T_x$. $Ti_3C_2T_x$ materials used in our report are composed of blocks with size of 1~3 μm, thus it displays a lower electrical conductivity than some reported large-sized $Ti_3C_2T_x$ materials (3~6 μm, ~2500 S cm^{-1}) [16], and shows the same order of magnitude compared with the reported $Ti_3C_2T_x$ materials with similar size (123 S cm^{-1} and 778 S cm^{-1}) [16-17].

The MDC material is anisotropic and exhibits good in-sheet conductivity. However, the electrical conductivity in the *c*-direction is relatively low due to the relatively long distance between the two adjacent nanosheets. Although the OMC is not highly conductive, it bridges the adjacent nanosheets in the MDC, and promotes the electron transportation in the *c*-direction. Thus, compared to the pure MDC, the MDC-OMC has a 3D cross-linked conductive networks and increased electrical conductivity. Similar observations were also published for other 2D material/OMC composites. For example, graphene oxide(GO)-resol derived rGO-porous carbon has a higher conductivity of 41.9 S cm^{-1} than the individual rGO (10~20 S cm^{-1}) and resol derived porous carbon (0.41 S cm^{-1}) [9]. The expanded graphite/OMC composite also shows lower resistance of 0.3638 Ω than the separated expanded graphite (0.4785 Ω) and OMC (0.4908 Ω) [18].

Supplementary Note 4. Detailed discussion on density of MDC and MDC-OMC electrode.

The MDC material has a layered structure with abundant in-plane and interlayer micropores. After rolling into the electrode film, the flake-to-flake restacking may lead to a little reduction in interlayer nanospace but the abundant in-plane micropores are still present. Therefore, the density of MDC electrode film is only about 0.52 g cm^{-3}.

The OMC material is formed in situ and is confined within the MDC interlayers, acting as rigid porous pillars and bridging the nanosheets. It has little influence on the interlayer of the MDC materials. In addition, the density of the OMC is higher than that of the MDC. As a result, the OMC would not only prevent the restacking of the layered nanosheets but also construct a more compact structure than in the MDC during the preparation of the electrode and electrochemical measurement. Thus, the MDC-OMC has a higher density of 0.83 g cm^{-3} than the pure MDC (0.52 g cm^{-3}).

The commercial activated carbon has a looser and more porous structure than the MDC-OMC due to the larger surface area (usually >1600 m^2 g^{-1}). In addition, the morphology of the MDC-OMC blocks is different from the commercial activated carbon, which also would have influence on the packing density.

Supplementary References

1 Simon, P. & Gogotsi, Y. Capacitive energy storage in nanostructured carbon–electrolyte systems. *Accounts of Chemical Research* **46**, 1094-1103 (2012).

2 Kang, D. M., Liu, M. L., Gu, J. J., Su, Y. S., Zhang, W. & Zhang, D. 'Egg-box'-assisted fabrication of porous carbon with small mesopores for high-rate electric double layer capacitors. *ACS Nano* **9**, 11225-11233 (2015).

3 Pachfule, P., Shinde, D., Majumder, M. & Xu, Q. Fabrication of carbon nanorods and graphene nanoribbons from a metal–organic framework. *Nature Chemistry* **8**, 718-724 (2016).

4 Zheng, X. Y., Lv, W., Tao, Y., Shao, J. J., Zhang, C., Liu, D. H., Luo, J. Y., Wang, D. W. & Yang, Q. H. Oriented and interlinked porous carbon nanosheets with an extraordinary capacitive performance. *Chemistry of Materials* **26**, 6896-6903 (2014).

5 Laušević, Z., Apel, P. Y., Krstić, J. B. & Blonskaya, I. V. Porous carbon thin films for electrochemical capacitors. *Carbon* **64**, 456-463 (2013).

6 Lei, Z., Lu, L. & Zhao, X. The electrocapacitive properties of graphene oxide reduced by urea. *Energy & Environmental Science* **5**, 6391-6399 (2012).

7 Yang, X., Cheng, C., Wang, Y., Qiu, L. & Li, D. Liquid-mediated dense integration of graphene materials for compact capacitive energy storage. *Science* **341**, 534-537 (2013).

8 Jiang, L., Sheng, L., Long, C. & Fan, Z. Densely packed graphene nanomesh-carbon nanotube hybrid film for ultra-high volumetric performance supercapacitors. *Nano Energy* **11**, 471-480 (2015).

9 Liu, R. L., Wan, L., Liu, S. Q., Pan L. X.; Wu, D. Q. & Zhao, D. Y. An interface-induced co-assembly approach towards ordered mesoporous carbon/graphene aerogel for high-performance supercapacitors. *Advanced Functional Materials* **25**, 526-533 (2015).

10 Byon, H. R., Lee, S. W., Chen, S., Hammond, P. T. & Shao-Horn, Y. Thin films of carbon nanotubes and chemically reduced graphenes for electrochemical micro-capacitors. *Carbon*

49, 457-467 (2011).

11 Sun, Y. *et al.* Highly conductive and flexible mesoporous graphitic films prepared by graphitizing the composites of graphene oxide and nanodiamond. *Journal of Materials Chemistry* **21**, 7154-7160 (2011).

12 Chmiola, J., Largeot, C., Taberna, P. -L., Simon, P. & Gogotsi, Y. Monolithic carbide-derived carbon films for micro-supercapacitors. *Science* **328**, 480-483 (2010).

13 Gao, P. C., Tsai, W. -Y., Daffos, B., Taberna, P. -L., Pérezd, C. R., Gogotsi, Y., Simon, P. & Favier, F. Graphene-like carbide derived carbon for high-power supercapacitors. *Nano Energy* **12**, 197-206 (2015).

14 Ding, B., Wang, J., Wang, Y., Chang, Z., Pang, G., Dou, H. & Zhang, X. G. A two-step etching route to ultrathin carbon nanosheets for high performance electrical double layer capacitors. *Nanoscale* **8**, 11136-11142 (2016).

15 Ling, Z. *et al.* Flexible and conductive MXene films and nanocomposites with high capacitance. *Proceedings of the National Academy of Sciences* **111**, 16676-16681 (2014).

16 Peng, Y. Y. *et al.* All-MXene (2D titanium carbide) solid-state microsupercapacitors for on-chip energy storage. *Energy & Environmental Science* **9**, 2847-2854 (2016).

17 Zhao, M. Q. *et al.* Flexible MXene/carbon nanotube composite paper with high volumetric capacitance. *Advanced Materials* **27**, 339-345 (2014).

18 Wang, L. *et al.* In situ intercalating expandable graphite for mesoporous carbon/graphite nanosheet composites as high-performance supercapacitor electrodes. *ChemSusChem* **5**, 2442-2450 (2012).
